# Variability in the Effects of Macroalgae on the Survival and Growth of Corals: The Consumer Connection

**DOI:** 10.1371/journal.pone.0079712

**Published:** 2013-11-15

**Authors:** Fabio Bulleri, Marine Couraudon-Réale, Thierry Lison de Loma, Joachim Claudet

**Affiliations:** 1 Dipartimento di Biologia, Università di Pisa, Pisa, Italy; 2 National Center for Scientific Research, USR 3278 CNRS-EPHE CRIOBE, University of Perpignan, Perpignan, France; 3 Laboratoire d’Excellence CORAIL, Perpignan, France; University of Pennsylvania, United States of America

## Abstract

Shifts in dominance from corals to macroalgae are occurring in many coral reefs worldwide. Macroalgal canopies, while competing for space with coral colonies, may also form a barrier to herbivorous and corallivorous fish, offering protection to corals. Thus, corals could either suffer from enhanced competition with canopy-forming and understorey macroalgae or benefit from predator exclusion. Here, we tested the hypothesis that the effects of the brown, canopy-forming macroalga, *Turbinaria ornata*, on the survival and growth of corals can vary according to its cover, to the presence or absence of herbivorous and corallivorous fish and to the morphological types of corals. Over a period of 66 days, two coral species differing in growth form, *Acropora pulchra* and *Porites rus*, were exposed to three different covers of *T. ornata* (absent *versus* medium *versus* high), in the presence or absence of fish. Irrespective of the cover of *T. ornata*, fish exclusion reduced mortality rates of *A. pulchra*. Following fish exclusion, a high cover of *T. ornata* depressed the growth of this branched coral, whilst it had no effect when fish species were present. *P. rus* suffered no damage from corallivorous fish, but its growth was decreased by high covers of *T. ornata*, irrespective of the presence or absence of fish. These results show that negative effects of *T. ornata* on some coral species are subordinate to those of fish predation and are, therefore, likely to manifest only on reefs severely depleted of predators. In contrast, space dominance by *T. ornata* may decrease the growth of other coral species regardless of predation intensity. In general, this study shows that susceptibility to predation may determine the severity of the effects of canopy-forming macroalgae on coral growth.

## Introduction

Coral reefs worldwide are threatened by multiple stressors (e.g., climate change, water quality degradation and over-exploitation; [1]). The decline in coral abundance can either occur gradually as a consequence of a chronic stress (e.g., fishing depletion of herbivore populations) or more abruptly, as a consequence of short-lasting perturbations (e.g., storms, predator outbreaks; [2]–[4]). Both types of stressors can interact and induce phase-shifts from coral- to macroalgal-dominated systems [5]. Identifying potential non-linear responses to increased level of a given stressor or a combination of stressors is fundamental for mitigating the worldwide degradation of coral reefs and sustaining coral reef resilience.

Independently from the causes promoting macroalgal dominance, monopolization of space by macroalgae can prevent coral recovery by suppressing settlement and recruitment of larvae [6], [7]. Macroalgae can reduce the fecundity, survival and growth of coral colonies through a variety of mechanisms, including shading, overgrowth, abrasion, the production of allelopathic agents or promoting infection by virulent bacteria [8]–[11]. In addition, macroalgae can influence corals indirectly, by altering the intensity of herbivory and predation. For instance, dense stands of *T. ornata*, a lower preference macroalga to herbivores, have been shown to provide refuge from grazing to macroalgal species that are readily consumed in open areas [12]. Thus, coral colonies occurring amidst canopy stands may face enhanced competition from understorey macroalgae. Recently, Hoey and Bellwood [13] have documented the avoidance of patches vegetated by *Sargassum* spp. by grazing and browsing fish, suggesting that alterations in herbivorous fish behavior could ultimately result in positive feed-backs that facilitate the persistence of macroalgal dominated states.

On the other hand, species susceptible to predation can benefit from the association with consumer-defended species (i.e., associational defense) when consumer pressure is high [14], [15]. Although competing with corals, some brown, canopy-forming macroalgae that colonize coral reefs (e.g., *Sargassum* spp. or *Turbinaria ornata*) enhance habitat complexity [12], [13] and may provide shelter from predation. On tropical reefs, branching corals (i.e., *Pocillopora* spp.) can be protected from the predatory seastar, *Acanthaster planci*, when associated to massive corals that either repel predators through their nematocyst defenses and crustacean guards [16], [17] or reduce the detection and access of prey by the predator [18]. Likewise, turf-forming and fleshy macroalgae (i.e., *Sargassum* spp.) have been found to protect juvenile corals from parrotfish damage [19]. Under these circumstances, the net effect of macroalgal canopies on corals could be the result of a trade-off between negative effects, due to direct competition and/or decreased control of macroalgae by herbivores and positive effects of decreased predation resulting from the exclusion of corallivore or omnivore species.

The outcome of such a trade-off would vary whether a coral species is mainly limited by competition *versus* predation. Negative effects of resource exploitation (i.e., space and light) or interference (i.e., abrasion, enhanced sedimentation, allelochemicals) competition with either canopy-formers or understory fleshy macroalgal species would prevail if a coral species is competitively weak and little susceptible to predation. In contrast, a coral species could benefit from the presence of macroalgal canopies when it is not competitively subordinate to macroalgae, but susceptible to predation.

Here, by means of a field experiment, we investigated how the effects of the canopy-forming macroalga, *Turbinaria ornata*, on corals can vary according to the presence/absence of consumers (both herbivores and corallivores). To the best of our knowledge, no study has investigated the interaction between *T. ornata* and corals. Coral susceptibility to predation largely varies among species and it is generally greater in branching than massive or mounding forms [18], [20]–[23]. Thus, we predicted that: i) in the presence of consumers (i.e., herbivores, omnivores and corallivores), the extent to which positive effects of *T. ornata* via sheltering from predators would counterbalance negative effects of direct competition or loss of macroalgal control by herbivores would vary according to coral susceptibility to predation; ii) in the absence of consumers, the effects of *T. ornata* on corals would be negative irrespective of their susceptibility to predation. In addition, given the importance of density-dependent mechanisms in regulating the sign and strength of species interactions [24], [25], we predicted that the effects of *T. ornata* on corals would vary according to its cover.

## Materials and Methods

This study was approved and conducted as part of ongoing research of the Centre de Recherches Insulaires et Observatoire del’Environnement (CRIOBE, USR 3278 CNRS-EPHE, LABEX ‘‘CORAIL’’). Data from this study will be made available upon request.

### Study site

**Figure 1:**
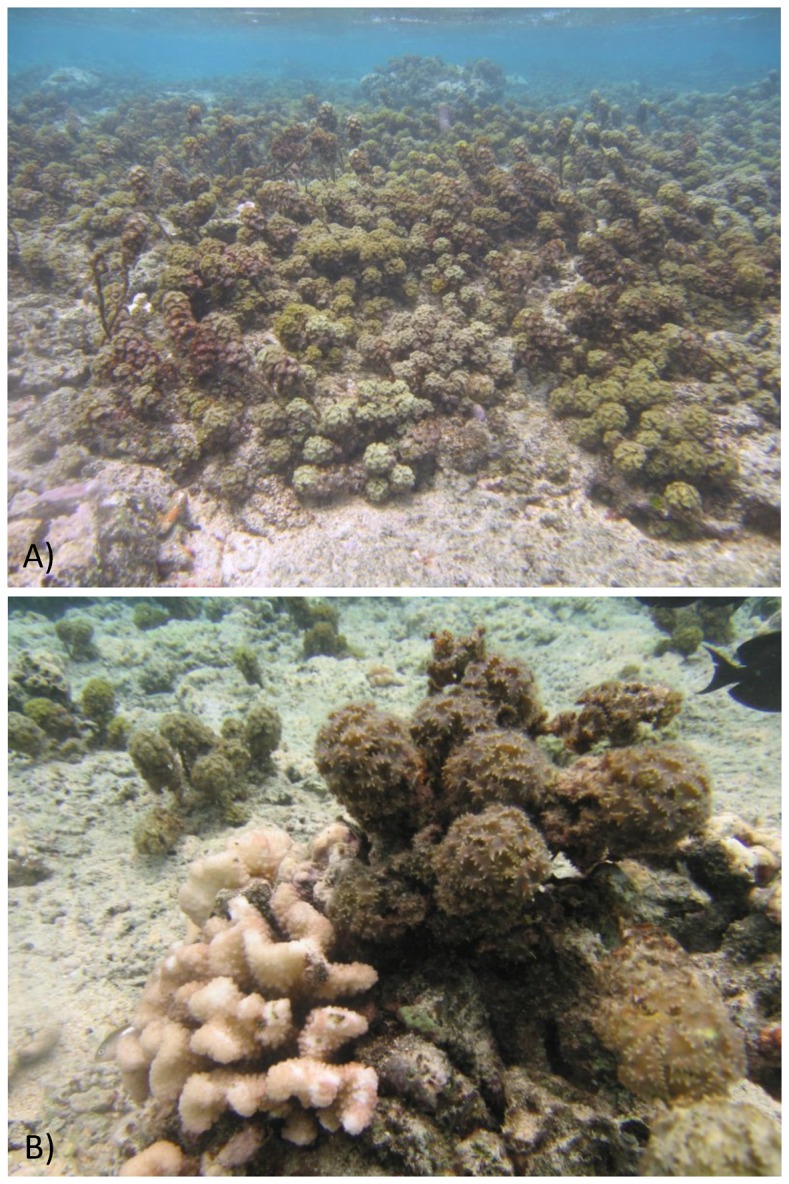
*Turbinaria ornata* in the mid-lagoon of Moorea’s north shore, French Polynesia. (A) Dense stands of *Turbinaria ornata* dominate substrata with low living coral cover. (B) A small sized *Pocillopora* sp. colony next to a thallus of *Turbinaria ornata*.

This study was conducted in the deepest part of the fringing reef of Moorea’s north shore, French Polynesia (17° 29’ 18.54’’ S; 149° 53’ 49.08’’ O), from February to May 2011. Over the past three decades, coral reefs on the island have experienced severe disturbance from cyclones, crown of thorns seastar (COTS) outbreaks and bleaching events that have promoted dominance by disturbance-tolerant corals, such as *Porites* spp. and *Pocillopora* spp. [23], [26], [27]. The macroalga, *Turbinaria ornata*, although not an alien species in French Polynesia, has become increasingly abundant in some lagoonal areas, possibly taking advantage of the large availability of suitable substrata for settlement (i.e., dead corals), loss of herbivores to over-fishing and nutrient inputs from terrestrial run-off [28], [29]. On the north shore of Moorea, dense stands of *T. ornata* are generally found in areas characterized by low covers of living corals and large availability of space, either bare or occupied by encrusting coralline macroalgae (Fig. 1A). In these areas, most coral colonies are small in size (Fig. 1B). *T. ornata* has mechanical (hard, rough tissue and rows of sharp spines on the blades) and chemical characteristics (production of phenolic compounds) that make it less preferable to herbivores [28]. A recent study by Rasher et al. [30] has demonstrated that, on Fijian reefs, brown macroalgae, including *T. ornata*, are almost exclusively consumed by unicornfishes (*Naso lituratus* and *N. unicornis*). In the back reefs of Moorea, *T. ornata* forms patches that vary both in plant density (from 10s up to several 100s of thalli per m^−2^; [31]) and extension (from 10s of cm^2^ to several m^2^; Bulleri et al. pers obs). In the Indo-Pacific, corallivorous fish preferentially target branching corals of the genera *Acropora* and *Pocillopora* [32]. Observations taken at our study site confirm the presence of a diverse fish assemblage, including corallivorous species, mostly belonging to genus *Chaetodon (*i.e., *C. citrinellus, C. vagabundus, C. lunulatus, C. auriga, C. lunula*), and several herbivorous species (List S1 in File S1).

### Experimental design

In order to mimic the spatial arrangement of coral colonies and *T. ornata* stands in degraded areas colonized by this macroalga, thirty-two 50×40×15 cm concrete slabs were deployed on rubble or sandy substrata surrounded by coral patches, between 1.5 and 3 m deep, ca. 10 days before the start of the experiment. Four slabs were randomly assigned to each of the 6 treatments generated by crossing 3 levels of cover of *T. ornata* (absent, medium and high cover) and 2 levels of consumers (presence *versus* absence of corallivores/herbivore fish). The remaining 8 slabs were used to check for potential artefact effects caused by the use of cages to exclude consumers. Slabs were wrapped with a coarse plastic gardening mesh (5 cm×5 cm mesh size). Thalli of *T. ornata* were fixed to the gardening mesh by means of plastic cable ties to generate three levels of cover: 1) 0 % (absent), 2) 40–50% (medium cover) and 3) 80–100% (high cover). Levels of covers included in the experiment encompassed the range of thallus density found in natural settings ([31]; authors’ personal observation). Thalli collected on nearby reef patches ranged between ∼ 25 and ∼ 40 cm in length and, thus, the number necessary to achieve a given level of cover slightly varied among slabs. In the case of slabs assigned to a medium cover of *T. ornata*, thalli were regularly distributed across the surface of the slab (i.e., they were not clumped).

Herbivore and corallivore fishes were excluded by means of cages made of plastic mesh (5 cm×5 cm). Cages were the same size of slabs aside (50 cm×40 cm) and 50 cm high. This height was chosen in order to reduce as much as possible alterations in the movement of *T. ornata*. A 20 cm wide lip was folded beneath the slab to ensure an effective sealing. Partial fences (2 sides removed, also referred to as half-cages), allowing fishes to move in and out of experimental plots, were used to control for potential artefact effects generated by caging. Because of time constraints, partial fences were established only at two levels of cover of *T. ornata* (absent and high cover; 4 slabs for each level of cover). Experimental slabs were visited on a daily basis (except for the case of adverse sea conditions) to maintain manipulative conditions. Detached thalli inside cages of *T. ornata* were, thus, replaced with newly collected individuals shortly after their dislodgement (≤ 2 days) and experimental covers maintained throughout the duration of the study. Fouling organisms were regularly removed from cages with a brush.

The effects of manipulative conditions were evaluated on two species of corals, *Acropora pulchra* and *Porites rus*, which differ markedly in their susceptibility to corallivory, tolerance to disturbance and growth rate [20], [21], [32], [33]. In general, massive and mounding forms are more tolerant to disturbance and less susceptible to corallivory than branching forms; the latter are, on the other hand, rapidly-growing and better competitors for space [18], [20]–[23]. The comparison between one branched (*A. pulchra*) and one sub-columnar species (*P. rus*) does not allow assessing formally to what extent differences in their response to manipulative conditions are due to the growth form or to other species-specific life-history traits. Thus, the differences between *A. pulchra* and *P. rus* documented by this study can be only indicative of the general response of branched and mounding forms to different combinations of macroalgal cover and presence/absence of herbivores and predators.

Nubbins provide an efficient way to manipulate corals and have been widely used in experimental studies [34], [35]. However, since there are uncertainties on how closely nubbins can reflect patterns of growth and mortality of juvenile colonies, their use should be considered as a relative test. We collected ∼ 50 thumb-sized nubbins from each of three separate genets (each colony was sampled in different sites to ensure that it did not belong to a same genet) of each of the two coral species tested, yielding a total of ∼ 150 nubbins for each species. Nubbins of *A. pulchra* were collected from the back-reef, as colonies of this species were scant in the mid-lagoon. The use of nubbins from different genets enabled to take into account, although not to test for, variation due to genetic diversity [35]. Nubbins were brought to the lab and kept in outdoor tanks (1.5 m^3^) with running, filtered seawater for 3–5 days to allow acclimatization. Nubbins were then mounted on green gardening plastic mesh, using epoxy putties (Z spar, Splash-zone and Veneziani S-Subcoat). Nubbins were kept in tanks for another 3–5 days after being mounted on the mesh and those showing signs of stress (e.g., bleaching), possibly due to the handling, transportation or contact with the epoxy putty, were not used for the field experiment. Just before translocation to the field, each nubbin was weighted using the buoyant mass technique [36]. Three nubbins of each species (one from each colony) were randomly allocated to each experimental slab (i.e., 6 nubbins × slab). Each nubbin was haphazardly positioned on the slab and fastened to the coarse gardening mesh by means of cable ties. Nubbins on a slab were generally spaced by 10s of cm. All of the 192 nubbins used in the experiment were transported and fixed to the experimental slabs on the same day (4 March 2011).

The damage due to fish biting was recorded ∼ 24 hours after the start of the experiment, using a semi-quantitative method. A score ranging from 0 to 4 was given to each nubbin according to the percentage of the coral surface damaged by fish predation (0: no damage; 1: 0–25%; 2: 25%–50%; 3: 50–75%; 4: more than 75%). Nubbins were retrieved from the field after 66 days (8 May 2011). Two pictures of each nubbin were taken from a randomly chosen angle and from a fixed distance using a digital camera. On a PC screen, we calculated the proportion of pixels occupied by algal turfs in relation to the total number of pixels enclosed within the coral profile, using the free software ImageJ (version 1.45, developed by W. Rasband, U. S. National Institutes of Health, Bethesda, Maryland, USA, http://imagej.nih.gov/ij/). Values of abundance of algal turfs were expressed as percentage cover and the mean value from the two pictures was used for statistical analysis. Algal turfs and encrusting corallines that had grown on nubbins, supporting mesh or on the epoxy were then carefully removed with tweezers and a plastic scrub before re-weighting nubbins. The standardized change in weight of each nubbin across the duration of the experiment was calculated as (*w_f_* – *w_i_*) / *wi*, where *w_i_* and *w_f_* are the initial and final weights, respectively. Dead corals were included in order to gain a comprehensive assessment of biomass variation due to consumption and growth.

### Statistical analyses

Coral mortality, net growth (i.e. decrease or increase in weight), overgrowth by algal turfs (expressed as percentage cover) and fish damage were analyzed, separately for *A. pulchra* and *P. rus*, by means of fully crossed ANOVAs including the factors Consumers (open *versus* caged; fixed) and *T. ornata* (absence *versus* medium cover *versus* high cover; fixed and crossed with Consumers), using the slab as the replicate (*n* = 4).

The same ANOVA models were used to test for potential artefact effects due to the presence of cages, but including two levels for each of the factors Consumers (open *versus* half-cages) and *T. ornata* (absence *versus* high cover). Homogeneity of variances was tested using Cochran’s test and data were transformed when necessary [37]. Student Newman Keuls (SNK) tests were used for *a posteriori* comparisons of the means.

## Results

**Figure 2:**
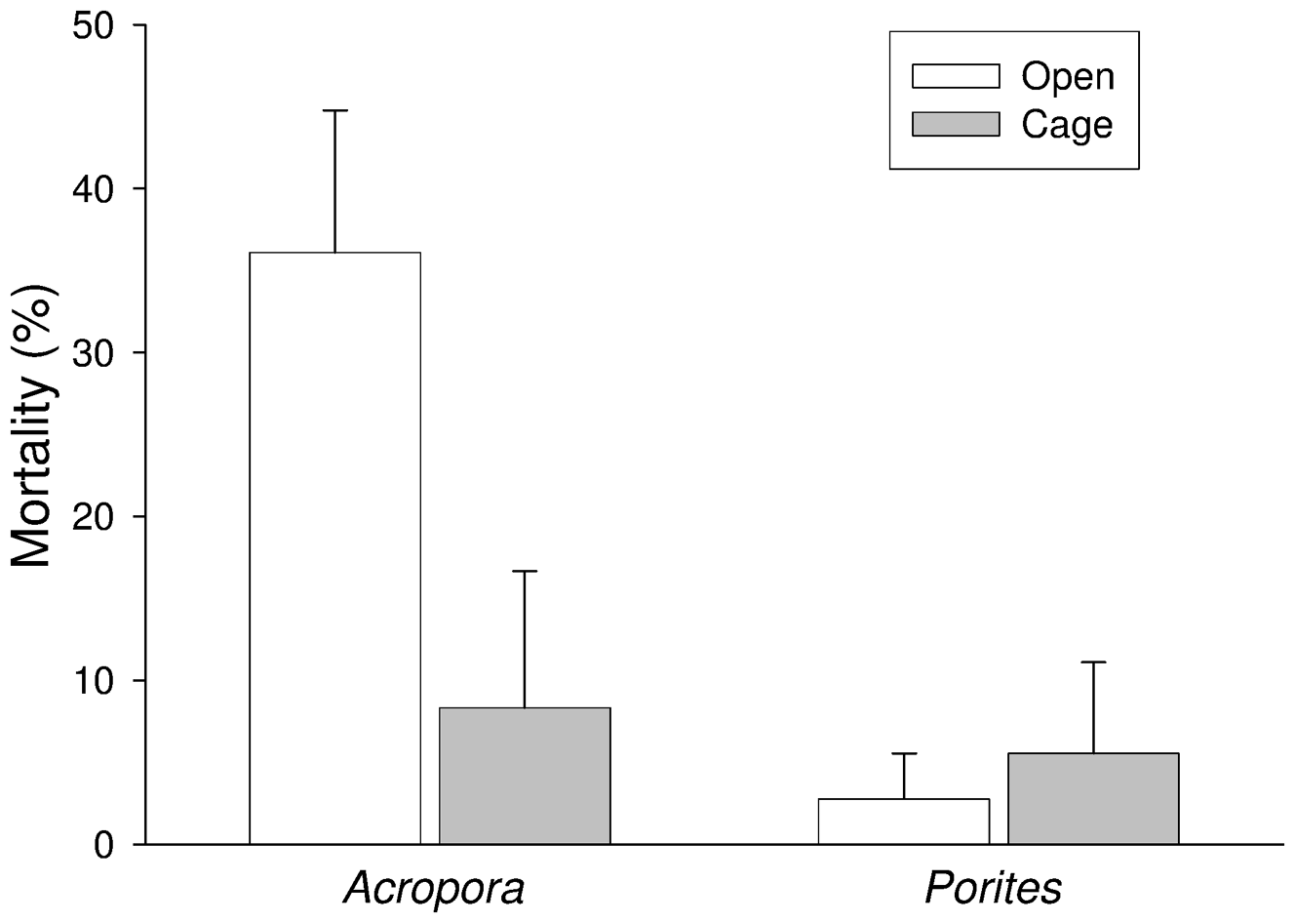
Percentage mortality (mean + 1SE) of *Acropora pulchra* and *Porites rus* in open and caged slabs. Data are pooled across slabs; *n* = 12.

The exclusion of fish significantly reduced mortality rates in *A. pulchra*, whilst it had no effect on *P. rus* (Table 1A, B; Fig. 2). The manipulation of the cover of *T. ornata* had no effect on coral mortality (Table 1 A, B). There was no artefact effect of cages on coral mortality (Table S1 in File S1).

**Table 1:** ANOVA on the effects of Consumers (present *versus* excluded) and *T. ornata* (absent *versus* medium cover *versus* high cover) on the mortality of A) *A. pulchra* and B) *P. rus*).

Source of variation		A) *A. pulchra*			B) *P. rus*	
	df	MS	*F*		MS	*F*
Consumers (C)	1	4629.630	5.56	*	46.296	0.20
*T. ornata* (T)	2	46.296	0.06		416.667	1.80
C × T	2	1990.741	2.39		46.296	0.20
Residual	18	833.333			231.481	
Transformation		None			None	
Cochran’s test		*P*>0.05			*P*<0.01	

* *P*<0.05.

**Figure 3:**
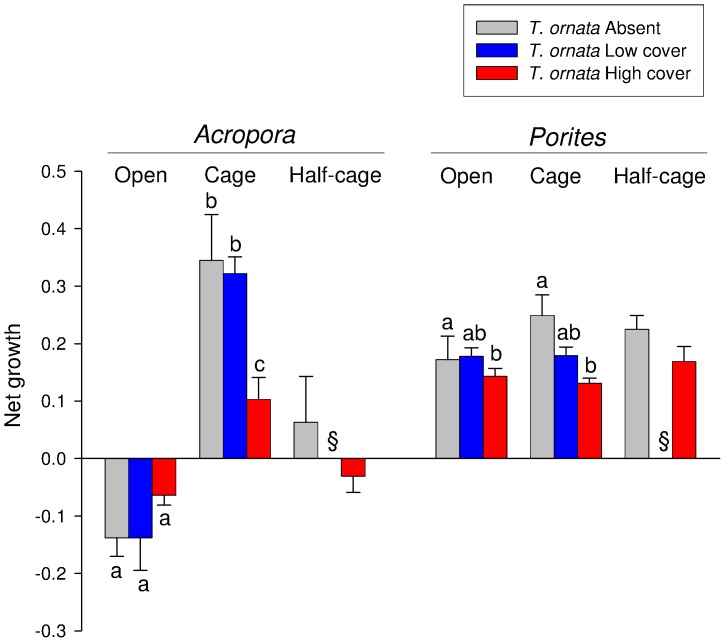
Net growth (mean + 1SE) of *Acropora pulchra* and *Porites rus* exposed to different combinations of consumers (open *versus* caged *versus* half-cages) and cover of *Turbinaria ornata* (absent *versus* medium cover *versus* high cover). Letters above bars illustrate the outcome of SNK (Student-Newman-Keuls) tests for the interaction Consumers × *T. ornata*; different letters indicate significant differences at *P*<0.05. Data are averages across slabs; *n* = 12. §  =  no data (the combination *T. ornata* medium cover and half cage was not included in the experiment design).

The effects of *T. ornata* on the growth of *A. pulchra* varied according to the presence/absence of fish (significant Consumers x *T. ornata* interaction; Table 2A). The weight of *A. pulchra* nubbins decreased on open slabs (Fig. 3). Although not statistically significant, there was a trend for a smaller decrease in the weight of *A. pulchra* when *T. ornata* was at a high cover than when it was absent or at a medium cover (Fig. 3). When fishes were excluded, there was an increment in the weight of *A. pulchra* that was significantly smaller when *T. ornata* was at a high cover (Fig. 3).

**Table 2:** ANOVA on the effects of Consumers (present *versus* excluded) and *T. ornata* (absence *versus* medium cover *versus* high cover) on the net growth of A) *A. pulchra* and B) *P. rus*.

Source of variation		A) *A. pulchra*			B) *P. rus*		
	df	MS	*F*		MS	*F*	
Consumers (C)	1	0.862	120.10	***	0.005	2.02	
*T. ornata* (T)	2	0.014	2.00		0.009	3.80	*
C × T	2	0.068	9.43	***	0.005	2.17	
Residual	18	0.007			0.002		
Transformation		None			None		
Cochran’s test		*P*<0.01			*P*<0.05		

*** *P*<0.001; * *P*<0.05.

The weight of *P. rus* increased over the study period and it was influenced by *T. ornata*, but not by the exclusion of consumers (Table 2B and Fig. 3). The SNK test indicated that the growth of *P. rus* on slabs with a high cover of *T. ornata* was significantly smaller than that on slabs without the macroalga (Fig. 3).

The variation in coral weight of *A. pulchra* differed between open slabs and half-cages (Table S2 in File S1). The analysis indicated that the gain in weight was greater for half cages than open slabs, consistently between covers of *T. ornata*. In contrast, there was no artefact of cages on the net growth of *P. rus* (Table S2 in File S1).

The cover of algal turf on corals was not influenced by the manipulation of the fish assemblage or *T. ornata* and did not differ between open slabs and half-cages (Tables S3 and S4 in File S1).

**Figure 4:**
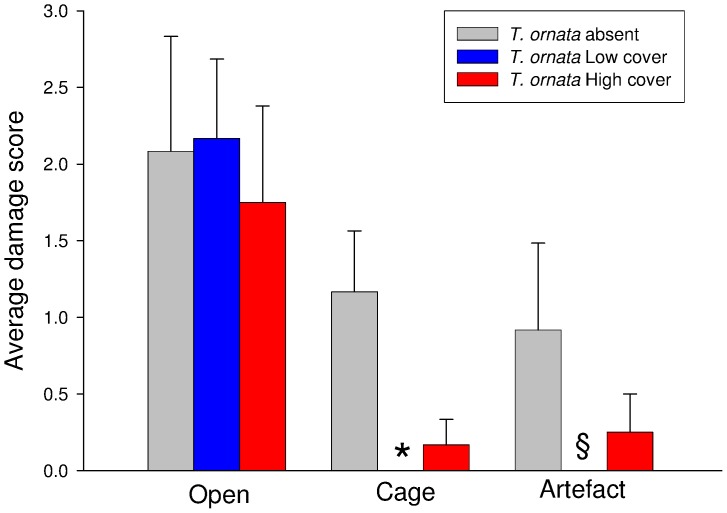
Damage score (mean + 1SE) experienced by *Acropora pulchra* individuals when exposed to different combinations of consumers (open *versus* cage *versus* half-cage) and *Turbinaria ornata* cover (absent *versus* medium *versus* high). Data are pooled across slabs; *n* = 12. *  =  0; §  =  no data (the combination *T. ornata* medium cover and half-cage was not included in the experiment design).

One day after deployment, none of the *P. rus* nubbins was damaged by corallivores. In contrast, a proportion varying between ∼ 92% and 75% of *A. pulchra* nubbins presented scars generated by fish bites in open slabs. The ANOVA (Consumers: MS  =  14.518, *F*_1, 18_  =  15.42, *P*<0.001; analysis on untransformed data, Cochran’s test: *P*>0.05) indicated that the severity of the fish-generated damage was significantly smaller in cages than in open plots (Fig. 4). *A. pulchra* nubbins damaged by fish on caged slabs suggest that cages were likely permeable to small-sized fish. There was no significant effect of *T. ornata* on the fish damage experienced by *A. pulchra*. The damage experienced by *A. pulchra* in half cages was significantly smaller than open slabs (Consumers: MS  =  7.111, *F*_1, 12_  =  5.30, *P*<0.05; analysis on untransformed data, Cochran’s test: *P*>0.05), suggesting that coral access in half-cages was reduced in respect to open slabs (Fig. 4).

## Discussion

*T. ornata* had no effect on the survival of either of the coral species tested. There were, in contrast, complex effects of *T. ornata* on coral growth that varied between coral species and according to the presence/absence of fish and to the macroalgal canopy cover.

In the presence of corallivorous and herbivorous fish, the net growth of *A. pulchra* was negative, irrespective of the cover of *T. ornata*. Thus, the enhancement of habitat structure generated by the macroalga, despite being previously shown to provide a refuge to fleshy macroalgae against herbivores [12], was not effective in reducing predation on branched corals. Within cages, dense stands of *T. ornata*, depressed the growth of *A. pulchra*. This clearly shows that, in open plots, negative effects of predation by corallivorous fish, by virtue of their greater intensity, masked those of competition with macroalgal canopies. Lack of differences in the growth of algal turfs on nubbins exposed to different covers of *T. ornata* indicate that negative effects of the macroalga on *A. pulchra* growth were likely direct (e.g., due to shading, abrasion, release of allelochemicals by *T. ornata* and not due to an enhancement of turf growth underneath the macroalgal canopy).

The net growth of *P. rus* was always positive and did not differ according to the presence or absence of fish. None of the *P. rus* nubbins on open slabs showed signs of fish biting, confirming previous evidence of a lower susceptibility of mounding forms to predation [18], [20]–[23]. The cover of algal turfs on *P. rus* was not influenced by *T. ornata*, suggesting that, like for branched corals, negative effects of large covers of the macroalga on *P. rus* growth were likely direct. Recently, several macroalgal species have been shown to cause major alterations to the microbial community associated to the mounding coral, *Porites astreoides*, ultimately decreasing its growth rate [38]. Similar mechanisms may be invoked to explain the negative effects of *T. ornata* on the growth of *P. rus*.

In the absence of *T. ornata*, the effects of excluding both corallivorous and herbivorous fish differed between the two species of coral tested. The exclusion of consumers reduced mortality rates and enhanced the growth of *A. pulchra*. This pattern, consistent with previous reports of a great susceptibility of branched corals to predation [18], [20], indicates that benefits of escaping predators were greater than disadvantages of losing macroalgal control by herbivores. Here, it is, however, worth stressing that the effects of predators and those of the herbivores may take place over different temporal scales. Effects of predation were, in fact, clearly evident just after 24 hrs from nubbin deployment, while those of grazers, due to their indirect nature (i.e., control of macroalgae competing with corals), may take considerable time to manifest, potentially longer than the time span of our study (i.e., 66 days).

The prevalence of direct positive effects of predator exclusion over indirect negative effects of herbivore exclusion has been documented for branching corals also in areas where the development of algal turfs was enhanced by farmerfish [35]. Thus, in Moorea, *A. pulchra* would be more limited by predation than competition with macroalgae [18], [35]. In a work recently conducted in Moorea, the removal of consumers did not influence mortality rates of recruits of *Acropora striata*, but caused a shift in the main cause of mortality, from predation to smothering by algal turfs [22]. Nubbins of *Acropora*, in virtue of their greater competitive ability in respect to settlers or recruits, might be less reliant on the control of macroalgae by herbivores.

In contrast, there was no effect of the exclusion of corallivorous and herbivorous fishes on mortality or growth rates of *P. rus*. Our results are in agreement with those of Gochfeld [21], who recorded little predation on *P. rus* when transplanted outside of damselfish territories. In addition, our results suggest that outside of damselfish territories, due to slower rates of macroalgal development, herbivores would play little role in regulating competitive interactions between *P. rus* and algal turfs. Susceptibility of *P. rus* to macroalgal overgrowth has been documented in artificial settings [39]. The relative isolation of experimental slabs from surrounding living substrata could have reduced the colonization ability of algal turfs, since vegetative propagation is the main mechanism for acquiring space of filamentous forms that compose turfing mats.

The use of cages created no artefacts on coral mortality, while it influenced their growth rates, in particular, that of *A. pulchra*. Worth to be noted is that the effects of half cages (2 sides removed) on coral growth were positive. This suggests that they could still limit the access of consumers to experimental slabs, as supported by the limited damage from fish biting on *A. pulchra* nubbins enclosed in half cages, after 1 day. Likely, our cages had no negative effects on coral growth through the alteration of key environmental variables, such as sedimentation rates, irradiance or water mass exchange. This would be in accordance with a study recently carried out in Moorea that documented no significant alteration in environmental conditions by cages of comparable mesh size [18]. Under these circumstances, artefact effects of cages are unlikely to impinge on the reliability of differences in coral growth emerged among different combinations of consumer presence/absence and covers of *T. ornata*.

At our study site, dense stands of *T. ornata* were generally found in areas characterized by low covers of living corals. Experimental slabs, deployed on sand or rubble substrata and surrounded by reef patches, mimicked the spatial arrangement of coral colonies and were, likely, exposed to levels of predation occurring in such degraded areas. In contrast, the relatively close association of the two coral species on experimental slabs did not reproduce their natural arrangement, as colonies of *A. pulchra* were scant in the mid-lagoon. At small spatial scales, coral susceptibility to predation and overall densities can influence relative predations rates [18], [40]. An intensification of predation rates on less preferred coral species has been documented on reefs characterized by the rarity of preferred prey species and low coral cover [41]. Here, *P. rus* suffered little predation damage, also suggesting that the attraction of corallivores by *A. pulchra* did not foster predation on this mounding species. Little predation on *P. rus* would indicate that consumer pressure on *A. pulchra* was not decreased through a dilution effect. Therefore, the relatively close association of the two coral species does not seem to have altered the natural functioning of the processes observed.

In summary, our results suggest that negative effects of canopy-forming macroalgae on branching corals growth are subordinate to those of consumers. Following Folt et al. [42], cumulative effects of predation and competition with canopy-forming macroalgae on coral growth may be classified as simple comparative effects, in that when the worst stressor is present, the weaker stressor has no additional impact. Within this conceptual framework, dense stands of macroalgal canopies would play a small role on corals on intact reefs, where *A. pulchra* mortality and growth is mainly regulated by predators. On the other hand, large stands of *T. ornata* are unlikely on relatively pristine reefs, given that the establishment and spread of this macroalga seems to be dependent upon habitat degradation [28], [30]. Our results suggest that levels of predation encountered on reefs heavily colonized by *T. ornata* would be sufficient to overwhelm any effect of the macroalga. Under these circumstances, we would argue that *T. ornata* is likely to affect branched coral growth only in areas where corallivorous fish assemblages have been severely depleted. The response of *P. rus* to experimental treatments suggests, in contrast, that negative effects of *T. ornata* on the growth of mounding corals are expected to be ubiquitous, as independent from consumers. More generally, this study shows that susceptibility to predation may regulate the severity of the effects of canopy-forming macroalgae on coral growth.

## Supporting Information

File S1 **Supporting information.**
(DOCX)

## References

[1] Hughes TP, Graham NAJ, Jackson JBC, Mumby PJ, Steneck RS (2010) Rising to the challenge of sustaining coral reef resilience. Trends Ecol Evol 25: 633–642. 20800316 10.1016/j.tree.2010.07.011

[2] Hughes TP (1994) Catastrophes, phase shifts, and large-scale degradation of a Caribbean coral reef. Science 265: 1547–1551. 17801530 10.1126/science.265.5178.1547

[3] Aronson RB, Precht WF (2001) White-band disease and the changing face of Caribbean coral reefs. Hydrobiologia 460: 25–38.

[4] Hughes TP, Rodrigues MJ, Bellwood DR, Ceccarelli D, Hoegh-Guldberg O, et al (2007) Phase shifts, herbivory, and the resilience of coral reefs to climate change. Curr Biol 17: 360–365. 17291763 10.1016/j.cub.2006.12.049

[5] McManus JW, Polsenberg JF (2004) Coral-algal phase shifts on coral reefs: ecological and environmental aspects. Prog Oceanogr 60: 263–279.

[6] McCook LJ, Jompa J, Diaz-Pulido G (2001) Competition between corals and algae on coral reefs: a review of evidence and mechanisms. Coral Reefs 19: 400–417.

[7] Birrell CL, McCook LJ, Willis BL (2005) Effects of algal turfs and sediment on coral settlement. Mar Pollut Bull 51: 408–414. 15757739 10.1016/j.marpolbul.2004.10.022

[8] River GF, Edmunds PJ (2001) Mechanisms of interaction between macroalgae and scleractinians on a coral reef in Jamaica. J Exp Mar Biol Ecol 261: 159–172. 11399272 10.1016/s0022-0981(01)00266-0

[9] Box SJ, Mumby PJ (2007) Effect of macroalgal competition on growth and survival of juvenile Caribbean corals. Mar Ecol Prog Ser 342: 139–149.

[10] Barott KL, Rodriguez-Brito B, Youle M, Marhaver KL, Vermeij MJA, et al (2011) Microbial to reef scale interactions between the reef-building coral *Montastraea annularis* and benthic algae. Proc Roy Soc B. 279: 1655–1664. 10.1098/rspb.2011.2155 PMC3282354 22090385

[11] Rasher DB, Stout EP, Kubanek J, Hay ME (2011) Macroalgal terpenes function as allelopathic agents against reef corals. Proc Natl Acad Sci USA 108: 17726–17731. 22006333 10.1073/pnas.1108628108 PMC3203809

[12] Bittick SJ, Bilotti ND, Peterson HA, Stewart HL (2010) *Turbinaria ornata* as an herbivory refuge for associate algae. Mar Biol 157: 317–323.

[13] Hoey AS, Bellwood DR (2011) Suppression of herbivory by macroalgal density: a critical feedback on coral reefs? Ecol Lett 14: 267–273. 21265975 10.1111/j.1461-0248.2010.01581.x

[14] Hay ME (1986) Associational plant defenses and the maintenance of species diversity: turning competitors into accomplices. Am Nat 128: 617–641.

[15] Stachowicz JJ (2001) Mutualism, facilitation, and the structure of ecological communities. Bioscience 51: 235–246.

[16] Glynn PW (1985) El Ninõ-associated disturbance to coral reefs and post-disturbance mortality by *Acanthaster planci* . Mar Ecol Prog Ser 26: 295–300.

[17] McKeon CS, Stier AC, McIlroy SE, Bolker BM (2012) Multiple defender effects: synergistic coral defense by mutualist crustaceans. Oecologia 169: 1095–1103. 22374368 10.1007/s00442-012-2275-2

[18] Kayal M, Lenihan HS, Pau C, Penin L, Adjeroud M (2011) Associational refuges among corals mediate impacts of a crown-of-thorns starfish *Acanthaster planci* outbreak. Coral Reefs 30: 827–837.

[19] Venera-Ponton DE, Diaz-Pulido G, McCook LJ, Rangel-Campo A (2011) Macroalgae reduce growth of juvenile corals but protect them from parrotfish damage. Mar Ecol Prog Ser 421: 109–115.

[20] Rotjan RD, Lewis SM (2008) Impact of coral predators on tropical reefs. Mar Ecol Prog Ser 367: 73–91.

[21] Gochfeld DJ (2010) Territorial damselfishes facilitate survival of corals by providing an associational defense against predators. Mar Ecol Prog Ser 398: 137–148.

[22] Penin L, Michonneau F, Carroll A, Adjeroud M (2011) Effects of predators and grazers exclusion on early post-settlement coral mortality. Hydrobiologia 663: 259–264.

[23] Lenihan HS, Holbrook SJ, Schmitt RJ, Brooks AJ (2011) Influence of corallivory, competition, and habitat structure on coral community shifts. Ecology 92: 1959–1971. 22073787 10.1890/11-0108.1

[24] Harper JL, White J (1974) The demography of plants. Annu Rev Ecol Syst 5: 419–463.

[25] Bulleri F, Cristaudo C, Alestra A, Benedetti-Cecchi L (2011) Crossing gradients of consumer pressure and physical stress on shallow rocky reefs: a test of the stress-gradient hypothesis. J Ecol 99: 335–344.

[26] Adjeroud M, Penin L, Carroll A (2007) Spatio-temporal heterogeneity in coral recruitment around Moorea, French Polynesia: Implications for population maintenance. J Exp Mar Biol Ecol 341: 204–218.

[27] Kayal M, Vercelloni J, Lison de Loma T, Bosserelle P, Chancerelle Y, et al (2012) Predator Crown-of-Thorns Starfish (*Acanthaster planci*) outbreak, mass mortality of corals, and cascading effects on reef fish and benthic communities. PLoS ONE 7(10): e47363. 23056635 10.1371/journal.pone.0047363 PMC3466260

[28] Stewart HL (2008) The role of spatial and ontogenetic morphological variation in the expansion of the geographic range of the tropical brown alga, *Turbinaria ornata* . Integr Comp Biol 48: 713–719. 21669827 10.1093/icb/icn028

[29] Adam TC, Schmitt RJ, Holbrook SJ, Brooks AJ, Edmunds PJ, et al (2011) G (2011) Herbivory, connectivity, and ecosystem resilience: response of a coral reef to a large-scale perturbation. PLoS ONE 6(8): e23717. 21901131 10.1371/journal.pone.0023717 PMC3162008

[30] Rasher DB, Hoey AS, Hay ME (2013) Consumer diversity interacts with prey defenses to drive ecosystem function. Ecology in press. 10.1890/12-0389.1 PMC3752656 23923498

[31] Stiger V, Payri CE (1999) Spatial and seasonal variations in the biological characteristics of two invasive brown algae, *Turbinaria ornata* (Turner) J. Agardh and *Sargassum mangarevense* (Grunow) Setchell (Sargassaceae, Fucales) spreading on the reefs of Tahiti (French Polynesia). Bot Mar 42: 295–306.

[32] Cole AJ, Pratchett MS, Jones GP (2008) Diversity and functional importance of coral-feeding fishes on tropical coral reefs. Fish Fish 9: 286–307.

[33] Lenihan HS, Adjeroud M, Kotchen MJ, Hench JL, Nakamura T (2008) Reef structure regulates small-scale spatial variation in coral bleaching. Mar Ecol Prog Ser 370: 127–141.

[34] Elahi R, Edmunds PJ (2006) Tissue age affects calcification in the scleractinian coral, *Madracis mirabilis* . Biol Bull 212: 20–28. 10.2307/25066577 17301328

[35] White J-SS, O'Donnell JL (2010) Indirect effects of a key ecosystem engineer alter survival and growth of foundation coral species. Ecology 91: 3538–3548. 21302826 10.1890/09-2322.1

[36] Davies PS (1989) Short-term growth measurements of corals using an accurate buoyant weighing technique. Mar Biol 101: 389–395.

[37] Underwood AJ (1997) Experiments in ecology: their logical design and interpretation using analysis of variance. Cambridge: Cambridge University Press. 504 p.

[38] Vega Thurber R, Burkepile DE, Correa AMS, Thurber AR, Shantz AA, et al (2012) Macroalgae decrease growth and alter microbial community structure of the reef-building coral, *Porites astreoides* . PLoS ONE 7(9): e44246. 22957055 10.1371/journal.pone.0044246 PMC3434190

[39] Dizon RM, Yap HT (2006) Effects of multiple perturbations on the survivorship of fragments of three coral species. Mar Pollut Bull 52: 928–934. 16458939 10.1016/j.marpolbul.2005.12.009

[40] Shantz AA, Stier AC, Idjadi JA (2011) Coral density and predation affect growth of a reef-building coral. Coral Reefs 30: 363–367.

[41] Burkepile DE (2012) Context-dependent corallivory by parrotfishes in a Carribean reef ecosystem. Coral Reefs 31: 111–120.

[42] Folt CL, Chen CY, Moore MV, Burnaford J (1999) Synergism and antagonism among multiple stressors. Limnol Oceanogr 44: 864–877.

