# Supplementary material for: Variability in the Effects of Macroalgae on the Survival and Growth of Corals: The Consumer Connection
Source: PLoS One. 2013 Nov 15;8(11):e79712. doi: 10.1371/journal.pone.0079712 (PMC3829870; doi:10.1371/journal.pone.0079712)
Supplement: File S1 — Supporting information. (DOCX) [file pone.0079712.s001.docx]

SUPPORTING INFORMATION

**List S1. Fish species observed at our study site and associated main diet.**

The species composition of fish assemblages at our study site was estimated by underwater visual census. The species present within a radius of 1 m around each slab were recorded during ten-minute long observations. The sampling was performed across three days.

*Abudefduf septemfasciatus* (Microalgae)

*Abudefduf sexfasciatus* (Zooplankton)

*Abudefduf sordidus* (Microalgae)

*Acanthurus nigricans* (Microalgae)

*Acanthurus nigrofuscus* (Microalgae)

*Acanthurus triostegus* (Microalgae)

*Anampses caeruleopunctatus* (Macroinvertebrates)

*Anampses melanurus* (Microinvertebrates)

*Balistapus undulatus* (Macroinvertebrates)

*Canthigaster amboinensis* (Microinvertebrates)

*Canthigaster solandri* (Microinvertebrates)

*Cephalopholis argus* (Nekton)

*Chaetodon auriga* (Microinvertebrates)

*Chaetodon citrinellus* (Corals)

*Chaetodon lunula* (Corals)

*Chaetodon lunulatus* (Corals)

*Chaetodon vagabundus* (Corals)

*Cheilinus chlorourus* (Macroinvertebrates)

*Cheilinus trilobatus* (Macroinvertebrates)

*Chlorurus microrhinos* (Microalgae - Corals)

*Chlorurus sordidus* (Microalgae)

*Chrysiptera leucopoma* (Microinvertebrates)

*Ctenochaetus striatus* (Microalgae)

*Dascyllus aruanus* (Zooplankton)

*Epibulus insidiator* (Macroinvertebrates)

*Epinephelus merra* (Nekton)

*Forcipiger flavissimus* (Microinvertebrates)

*Gnathodentex aureolineatus* (Macroinvertebrates)

*Gomphosus varius* (Macroinvertebrates)

*Halichoeres marginatus* (Macroinvertebrates)

*Halichoeres ornatissimus* (Macroinvertebrates)

*Monotaxis grandoculis* (Macroinvertebrates)

*Mulloidichthys flavolineatus* (Macroinvertebrates)

*Mulloidichthys vanicolensis* (Macroinvertebrates)

*Myripristis berndti* (Macroinvertebrates - Zooplankton)

*Myripristis violacea* (Zooplankton)

*Naso lituratus* (Macroalgae)

*Ostracion meleagris* (Macroinvertebrates)

*Parapercis millepunctata* (Macroinvertebrates)

*Parupeneus cyclostomus* (Nekton)

*Parupeneus insularis* (Macroinvertebrates)

*Parupeneus multifasciatus* (Macroinvertebrates)

*Rhinecanthus aculeatus* (Macroinvertebrates)

*Scarus altipinnis* (Microalgae)

*Scarus oviceps* (Microalgae)

*Scarus psittacus* (Microalgae)

*Siganus spinus* (Microalgae)

*Stegastes nigricans* (Microalgae)

*Stethojulis bandanensis* (Microinvertebrates)

*Stethojulis strigiventer* (Microinvertebrates)

*Thalassoma hardwicke* (Microinvertebrates)

*Thalassoma quinquevittatum* (Microinvertebrates)

*Zebrasoma scopas* (Microalgae)

*Zebrasoma veliferum* (Microalgae)

Table S1. ANOVA on the effects of caging artefacts (open *versus* half-cages) and *T. ornata* (absent *versus* high cover) on the mortality of A) *A. pulchra* and B) *P. rus*).

| Source of variation |  | **A) *A. pulchra*** | |  |  | **B) *P. rus*** | | |
| --- | --- | --- | --- | --- | --- | --- | --- | --- |
|  | df | MS | *F* | |  | MS | *F* |  |
| Consumers (C) | 1 | 69.444 | 0.05 |  |  | 0.000 | 0.00 |  |
| *T. ornata* (T) | 1 | 69.444 | 0.05 |  |  | 0.000 | 0.00 |  |
| C × T | 1 | 3402.778 | 2.33 |  |  | 277.778 | 2.00 |  |
| Residual | 12 | 1458.333 |  |  |  | 138.889 |  |  |
| Transformation |  | None |  |  |  | None |  |  |
| Cochran’s test |  | *P* > 0.05 |  |  |  | *P* > 0.05 |  |  |

Table S2. ANOVA on the effects of caging artefacts (open *versus* half-cages) and *T. ornata* (absent *versus* high cover) on the net growth of A) *A. pulchra* and B) *P. rus*). * *P* < 0.05.

| Source of variation |  | **A) *A. pulchra*** | |  |  | **B) *P. rus*** | | |
| --- | --- | --- | --- | --- | --- | --- | --- | --- |
|  | df | MS | *F* | |  | MS | *F* |  |
| Consumers (C) | 1 | 0.053 | 6.39 | * |  | 0.007 | 2.52 |  |
| *T. ornata* (T) | 1 | 0.000 | 0.00 |  |  | 0.006 | 2.01 |  |
| C × T | 1 | 0.026 | 3.06 |  |  | 0.001 | 0.45 |  |
| Residual | 12 | 0.008 |  |  |  | 0.003 |  |  |
| Transformation |  | None |  |  |  | None |  |  |
| Cochran’s test |  | *P* < 0.01 |  |  |  | *P* < 0.01 |  |  |

Table S3. Analysis of variance on the effects of Consumers (present *versus* excluded) and *T. ornata* (absence *versus* low cover *versus* high cover) on algal turf overgrowth in A) *A. pulchra* and B) *P. rus*.

| Source of variation |  | **A) *A. pulchra*** | |  |  | **B) *P. rus*** | | |
| --- | --- | --- | --- | --- | --- | --- | --- | --- |
|  | df | MS | *F* | |  | MS | *F* |  |
| Consumers (C) | 1 | 13.018 | 1.07 |  |  | 0.810 | 0.55 |  |
| *T. ornata* (T) | 2 | 11.666 | 0.96 |  |  | 3.882 | 2.61 |  |
| C × T | 2 | 11.666 | 0.96 |  |  | 0.364 | 0.25 |  |
| Residual | 18 | 12.117 |  |  |  | 1.485 |  |  |
| Transformation |  | None |  |  |  | Ln(x+1) | | |
| Cochran’s test |  | *P* < 0.01 |  |  |  | *P* > 0.05 |  |  |

Table S4. Analysis of variance on the effects of caging artefacts (open *versus* half-cages) and *T. ornata* (absent *versus* high cover) on algal turf overgrowth in A) *A. pulchra* and B) *P. rus*.

| Source of variation |  | **A) *A. pulchra*** | |  | **B) *P. rus*** | | |
| --- | --- | --- | --- | --- | --- | --- | --- |
|  | df | MS | *F* | | MS | *F* |  |
| Consumers (C) | 1 | 222.495 | 1.88 |  | 5.731 | 0.25 |  |
| *T. ornata* (T) | 1 | 232.062 | 1.96 |  | 27.922 | 1.20 |  |
| C × T | 1 | 549.289 | 4.65 |  | 11.637 | 0.50 |  |
| Residual | 64 | 118.241 |  |  | 23.327 |  |  |
| Transformation |  | None |  |  | None |  | |
| Cochran’s test |  | *P* < 0.01 |  |  | *P* > 0.05 |  | |
